# A process optimization for bio-catalytic production of substituted catechols (3-nitrocatechol and 3-methylcatechol

**DOI:** 10.1186/1472-6750-10-49

**Published:** 2010-06-30

**Authors:** Dhan Prakash, Janmejay Pandey, Bhupendra N Tiwary, Rakesh K Jain

**Affiliations:** 1 Institute of Microbial Technology (CSIR), Sector 39-A, Chandigarh160036, India; 2 Department of Biotechnology, Guru Ghasidas Viswavidyalaya, Bilaspur (C.G.) 495009, India

## Abstract

**Background:**

Substituted catechols are important precursors for large-scale synthesis of pharmaceuticals and other industrial products. Most of the reported chemical synthesis methods are expensive and insufficient at industrial level. However, biological processes for production of substituted catechols could be highly selective and suitable for industrial purposes.

**Results:**

We have optimized a process for bio-catalytic production of 3-substituted catechols viz. 3-nitrocatechol (3-NC) and 3-methylcatechol (3-MC) at pilot scale. Amongst the screened strains, two strains viz. *Pseudomonas putida *strain (F1) and recombinant *Escherichia coli *expression clone (pDTG602) harboring first two genes of toluene degradation pathway were found to accumulate 3-NC and 3-MC respectively. Various parameters such as amount of nutrients, pH, temperature, substrate concentration, aeration, inoculums size, culture volume, toxicity of substrate and product, down stream extraction, single step and two-step biotransformation were optimized at laboratory scale to obtain high yields of 3-substituted catechols. Subsequently, pilot scale studies were performed in 2.5 liter bioreactor. The rate of product accumulation at pilot scale significantly increased up to ~90-95% with time and high yields of 3-NC (10 mM) and 3-MC (12 mM) were obtained.

**Conclusion:**

The biocatalytic production of 3-substituted catechols viz. 3-NC and 3-MC depend on some crucial parameters to obtain maximum yields of the product at pilot scale. The process optimized for production of 3-substituted catechols by using the organisms *P. putida *(F1) and recombinant *E. coli *expression clone (pDTG602) may be useful for industrial application.

## Background

Precursors are essential starting material for the production of a large number of fine chemicals, antibiotics and flavor compounds. Catechols and their derivatives have wide industrial applications as they serve as precursors for large-scale synthesis of industrial and agricultural products [1,2]. Some catechols and their derivatives are used in manufacturing of synthetic flavors such as vanillin and fragrance [3]. A total of ~50% catechols are used in the synthesis of pesticides such as carbofuran, propouxur etc. [4]. They are also used as reagents in photography, polymerization inhibitors, dying industries for the production of rubber and plastic [5]. Substitutions on the 3 position of catechols are of particular interest due to their potential use as precursors in the synthesis of a wide range of pharmaceuticals including adrenergic catecholamines, biogenic amines and L-DOPA (used in hospitals for anesthesia) [6,7]. There are only a few world wide sources of 3-NC production and it is highly priced at app. $20,000/g [8]. Chemical synthesis of 3-substituted catechols is difficult due to low regio- selectivity and low stability of the product (the product is oxidized faster than the substrate). Stereo-selectivity in chemical synthesis of these catechols is also inadequate. In addition, chemical synthesis involves multiple reaction steps and consequently the recovery of chemically synthesized catechols on an industrial scale is rather poor [1,7]. Chemical synthesis of catechols also results a mixture of both 3- and 4- substituted catechols thereby increasing the cost of downstream processing enormously [9,10].

It has been established that microorganisms are capable of carrying out many enzymatic reactions that can be highly selective and/or specific and therefore, can be utilized for the production of 3-substituted catechols [11-13]. Till date there are only a few reports on bio-catalytic production of substituted catechols. Chae et al. [14], applied aqueous/organic two-phase reaction system for the production of catechols by using immobilized resting cells of *P. putida*. In another report, Boshoff et.al [15], investigated accumulation of catechols using membrane-immobilized polyphenol oxidase with two sequential cresolase and catecholase reactions. Vardar et al. [8], demonstrated formation of substituted nitrocatechol by oxidation of nitrobenzene using a toluene-o-xylene monooxygenase protein of *Pseudomonas stutzeri *OX1. The major drawback suggested by the above studies are that, although a number of compounds are transformed or degraded via formation of catechols as a major intermediate, however, their accumulation is quite low. Further, there are virtually no reports in our knowledge available on bio-catalytic production of 3-substituted catechols at pilot scale.

The present work describes a process optimization for the production of 3-substituted catechols at pilot scale using *P. putida *(F1) and recombinant *E. coli *expression clone (pDTG602). The process was initially optimized for the production of 3-substituted catechols on laboratory scale and subsequently at pilot scale studies were also performed using 2.5 liter bioreactors to maximize the product yields. The results obtained during the present study indicate the purity/selectivity and increased products yield as well.

## Methods

### Biological and chemical materials

Strain *P. putida *F1 (ATCC No. 700007) was procured from American Type Culture Collection (ATCC) , USA. A toluene transforming *E. coli *recombinant expression clone (pDTG602) was a generous gift from Prof. G. J. Zylstra (Department of Biochemistry and Microbiology, Rutgers Universtiy, NJ. USA). 3-Nitrophenol, toluene, 3-nitrocatechol, 3-methylcatechol and *p*-toluidine were obtained from Sigma Aldrich Corp. (St. Louis, MO. USA). All other chemicals used were of highest purity grade available locally.

### Bacterial strains and growth conditions

During the present study the soil samples from pesticide contaminated sites were screened for microorganisms capable of degrading/transforming 3-NP and toluene isolated by enrichment-culture techniques. Briefly, 1 g of fresh contaminated soil resuspended in 50 ml minimal media (MM) supplemented with 10 mM of glucose and lower selective concentration (0.5 mM) of 3-NP and vapors of toluene, incubated at 30°C overnight at 150 rpm. The isolated organisms were grown in enriched source medium such as Nutrient broth (NB) or Luria Broth (LB) or low nutrients MM containing 3-NP and/or toluene as the biotransformation substrates. The MM was used had the following composition (per liter) Na_2_HPO_4_: 4.0 g; KH_2_PO_4_: 2.0 gm; MgSO_4_: 0.8 gm; (NH_4_)_2_SO_4_: 0.8 gm; yeast extract; 1.00 gm and trace element solution Al(OH)_3_: 0.1 gm; SnCl_2_: 0.5 gm; KI:0.5 gm; LiCl: 0.05 gm; MnSO_4_.4H_2_O: 0.08 gm; H_3_BO_3_: 0.5 gm; ZnSO_4_.7H_2_O: 0.1 gm; CoCl_2_.6H_2_O: 0.1 gm; NiSO_4_.6H_2_O: 0.1 gm; BaCl_2_:0.05 gm; (NH_4_)_6_Mo_7_O_24_.4H_2_O: 0.05 gm. The MM was prepared as described earlier by Prakash et al. [16]. Incubations were carried out at optimized temperature, pH and substrate concentration with aeration. Bacterial growth was determined by monitoring the OD_600 _spectrophotometrically (Lambda EZ-201 UV-Vis Spectrophotometer, Perkin-Elmer, USA).

### Chromogenic assay for detection of catechols

Bacterial isolates obtained from the enrichment culture technique were screened for the production of 3-substituted catechols including wild type strains *P. putida *F1 and recombinant *E. coli *expression clone (pDGT602) using a chromogenic plate based assay as described by Parke, [17]. The overnight grown seed cultures were inoculated on MM agar plates containing 3-NP and toluene as biotransformation substrates. The inoculated plates were incubated at 30°C for 24 h. The aliquots of 1-2 ml of *p*-toluidine (1 M stock solution of *p*-toluidine prepared in N, N-dimethylformamide) were spread on the O/N grown plates in presence of 0.5 M solution of ferric chloride. The development of deep red-brown precipitates on the plates were indicating the formation of substituted catechols in the medium. The organisms found to be positive for above chromogenic assay were selected for further process optimization for the production of 3-substituted catechol at pilot scale.

### Enzymatic assay for biotransformation

Cell extracts of *P. putida *F1 and recombinant *E.coli *expression clone were prepared by sonification of the cells for 10 min and the crude extract was centrifuged 10,000 rpm at 4°C for 20 min as described by Kieboom et al. [18]. Protein was determined according to the method as described by Bradford, [19]. The study of enzyme toluene dioxygenase assay by *P. putida *F1 from 3-NP to 3-NC was performed as described by Gibson et al., de Bont et al. [20,21].The volume of reaction mixture contained 3-NP (5 mM), 50 mM potassium phosphate buffer (pH 7.0), cell extract (30-40 mg of protein) in a final volume of 1 ml. The reaction was initiated by addition of substrate and reaction mixture was scanned 250-450 nm after every 2 min using a spectrophotometer (Perkin Elmer Lambda EZ201 UV/Vis) at room temperature. The activity of toluene dioxygenase was measured by increasing absorbance at A_max _292 nm indicating the formation of 3-NC. However, the activity of toluene dioxygenase and *cis*-dihydrodiol dehydrogenase from toluene to 3-MC by clone pDTG602 were carried out by radioactive toluene dioxygenase assays using radiolabelled [^14^C] toluene (500 μM) in dimethylformamide (specific activity 74.4 μCi/μM). The reaction mixture contained cell extract (20-40 mg of protein), 2 mM NADH, 150 μM ferrous sulphate and 2.5 μl of [^14^C] toluene in a final volume of 1 ml by 50 mM Bis- Tris propane HCl (pH 6.8) as described by Gibson et al. [20]. Reaction was initiated by addition of [^14^C] toluene. After 10 min of incubation, 100 μl of reaction mixture was applied to a piece of plastic-backed silica (15 cm by 20 cm) to absorbed metabolite and the sample was dried for 20 min to remove volatile [^14^C] toluene. The amount of radiolabelled metabolite was formed measured by scintillation counting (Beckman LS6800 scintillation counter, USA). The formation of reaction products were quantified and identified using authentic standard of 3-NC and 3-MC. The solutions were extracted twice at acidic pH 2.0 (6N HCl) with ethyl acetate. The reaction product 3-NC formed was rationally correlated with consumption of the substrate (3-NP) and identified by HPLC. However, the formation of 3-MC was quantified by measuring of a total of 84% of radioactivity (878,000 DPM) by counting nonvolatile^14^C-labeled metabolites. The transformed metabolite was further identified by GC-MS with EI using authentic standard of 3-MC.

### Process optimization for biotransformation

To carry out the transformation process at pilot scale various parameters were initially optimized at laboratory shake flask scale (250 ml volume) in order to produce 3- substituted catechols in the medium using *P. putida *strains F1 and the recombinant *E. coli *expression clone pDTG602.

### Optimization of different conditions

Different media such as NB, LB and MM with different concentration of glucose and sodium succinate (5 mM, 10 mM and 20 mM), varying pH (6.5, 7.5, 8.0 and 8.5) and incubation temperature (28°C, 30°C, 35°C and 37°C) were tested at laboratory scale for optimum production of 3-NC and 3-MC. Similarly, different range of aeration (180 rpm, 190 rpm, 200 rpm, 210 rpm and 220 rpm) were tested. Varying concentration of 3-NP (.5 mM, 1 mM, 2 mM, 3 mM, 4 mM, 5 mM and 6 mM) were also tested. Toluene was provided to the growing cultures in the form of saturated vapors. A range of inoculums size (1%, 1.5%, 2.0%, 2.5%, 3.0%, 3.5%, 4.0%, 4.5% and 5.0%) were also attempted to obtain optimum yields of the desired products.

### Effect of induced growth on transformation

The effect of induction on transformation was also optimized using large cell mass of strains *P. putida *F1 and recombinant *E. coli *expression clone (pDTG602). For pre-induction, cells of strains F1 was grown in 250 ml of MM supplemented with 15 mM glucose. However, the cells of clone (pDTG602) was grown in LB (OD600 ~1.0). The pre-grown cells were then re-suspended in 50 ml MM containing 5 mM of 3-NP or toluene saturated vapors as biotransformation substrate in order to evaluate the effect of induction on transformation efficiency.

### Downstream extraction

Down stream extraction of the transformed products 3-NC and 3-MC was carried out as described by Husken et al. [22]. Briefly, process samples (~10 ml) were collected at different time intervals from the transformation broth. The time points selected for samples collection were rationalized by chromogenic spot test (200 μl sample + 100 μl *p*-toluidine + 100 μl of ferric chloride) in microtiter plate. The intensity of the visible red- brown color and concentrations of 3-substituted catechols formation were measured at 510 nm using standard calibration curve of 3-NC and 3-MC. Collected samples were centrifuged to separate the cell mass and supernatant. The medium supernatants were solvent extracted twice with equal volumes of ethyl acetate/diethyl ether/octanol to separate the organic components of the samples from non-organic contents. Finally, the collected organic component of the samples were evaporated to dryness under nitrogen flow using TurboVap-II (Caliper Life Sciences, USA) and re-suspended in methanol for chromatographic analysis.

### Qualitative and quantitative analysis

Qualitative and quantitative analyses of the transformed products were performed using different analytical techniques such as Thin Layer Chromatography (TLC), Gas Chromatography (GC) and High performance Liquid Chromatography (HPLC). For TLC extracted samples were resuspended in adequate amount of methanol and analyzed on a silica gel coated TLC aluminum 60 F_254 _plates (20 × 20 cm thickness, E. Merck, Germany). The solvent system used was toluene: ethyl acetate: benzene in a ratio of 60:35:5. The TLC sheets were analyzed under near and far UV electromagnetic radiation. R*f *values of individual spots were calculated according to the standard method and compared with the R*f *values of the known compounds. GC analysis was carried out using flame ionization detector (FID) and a HP-1 (100% dimethyl polysiloxane) capillary column (30 m × 0.25 mm) (Perkin Elmer, MA, USA). The GC parameters used were as follows: injector temperature, 250°C; oven temperature, gradient 150- 230°C over 15 min; and detector temperature, 200°C. HPLC analysis was performed on Water 600 (Milford, MA, USA) equipped with a 996 photodiode array detector operated at 190-600 nm. Separation was carried out with C-18 reverse phase column (5 um, 4.6×250 nm; waters spherisorb ODS2) using as mobile phase of 0.1% tri-fluroacetic acid (TFA) in methanol: water (1:1) for 3-MC and 0.1% glacial acetic acid in methanol: water (1:1) for 3-NC at an isocratic flow rate of 1.5 ml/min.

### Pilot scale biotransformation

**Figure 1:**
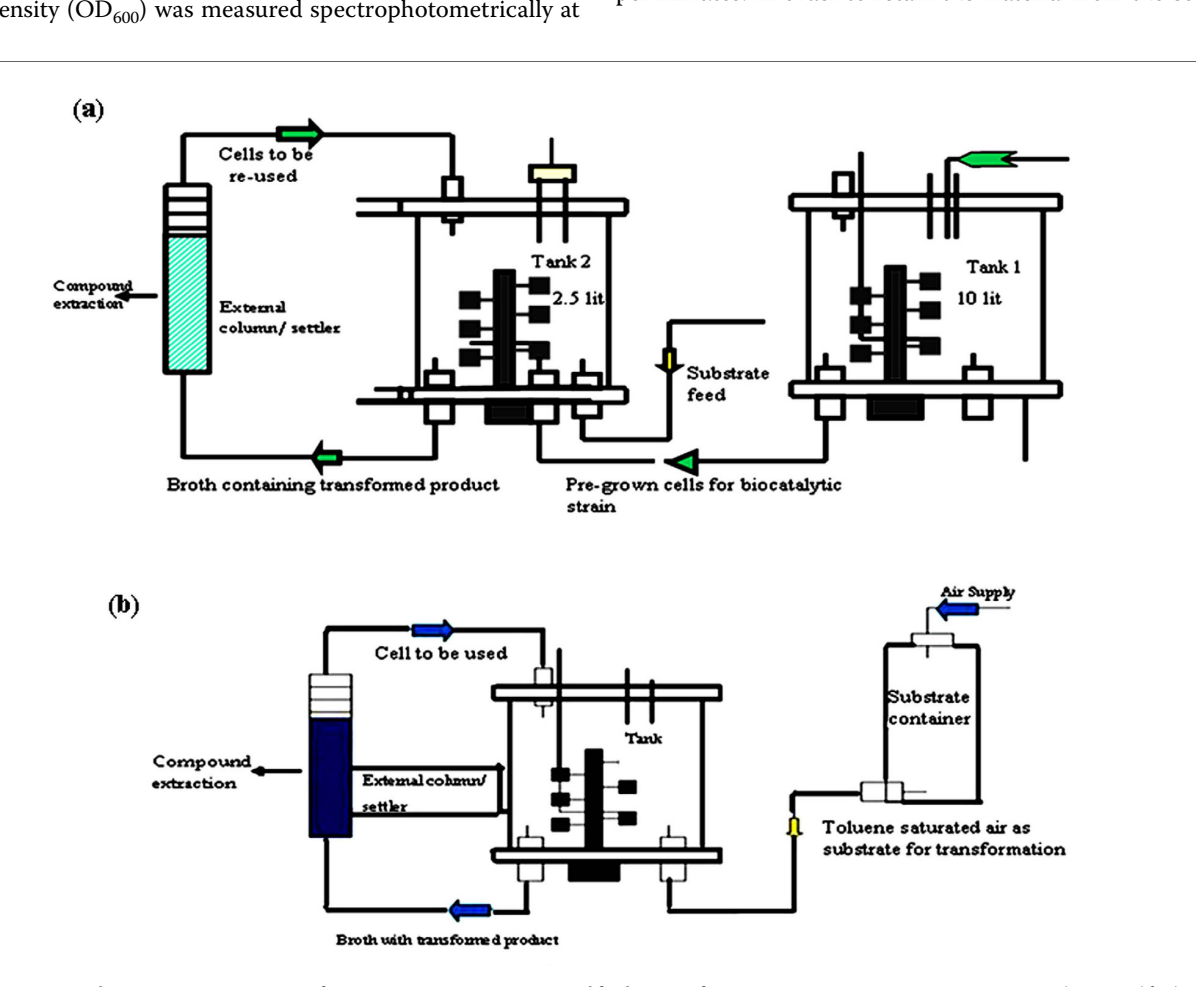
**Schematic representation of bioreactor/recovery loop used for biotransformation**. **(a) **Two-step bioreactor/recovery loop used for biotransformation of 3-nitrophenol to 3-nitrocatechol by strain *P. putida *F1. **(b) **Single- step bioreactor/recovery loop used for biotransformation of toluene to 3-methylcatechol by *E. coli *expression clone (pDTG602).

For the biotransformation of 3-NP to 3-NC, a two step feed batch bioreactor/recovery loop was optimized at pilot scale (Figure 1a). Pre-grown cells of the catalytic strain *P. putida *F1 was prepared by overnight growth in 10 liter NB at 30°C with aeration provided at a rate of 1 vvm compressed and on-line filter sterilized air. Optical density (OD_600_) was measured spectrophotometrically at regular time intervals and finally bioreactor was harvested at an OD_600 _was ~ 2.0 or cell densities reached between 5-10 g CDWL^-1^. The pre-grown biomass was separated from the growth medium by centrifugation at 8500 rpm for 10-20 minutes. The pelleted cells were re-suspended in 50 ml MM and then inoculated in 2.5 liter biotransformation broth containing MM with 15 mM of glucose. The process of biotransformation was allowed to proceed for 48 h at 30°C. The pH, temperature and dissolved oxygen content of the transformation broth were monitored during the process. The substrate 3-NP was feed at the constant rate of (0.12 mole/l^-1^/h^-1^) and product removed continuously by external adsorbent column containing resin Amberlite ™ XAD-4 attached to the bioreactor in order to reduced the bioreactor toxicity of the substrate and product. The cell containing broth was continuously circulated between bioreactor and external loop at a flow rate of 0.06 to 0.08 total volume changes per minutes. In order to retain the material from the column, lower end of the column was equipped with filter cloth and upper end with a wire mesh made up stainless steel (diameter 5.5 cm, mesh width 315 um, wire diameter 0.2 mm). The mesh filters allowed to pass bacterial cultures freely and retain the XAD-4. The cells and culture supernatant was separated from XAD-4 by filtration (Whatman 3 mm × 15 cm) and analyzed elute and broth by HPLC as described by Held et al. and Robinson et al. [7,23].

For the bioconversion of toluene to 3-MC, a single step batch bioreactor was optimized (Figure 1b). An overnight pre-grown induced culture of pDTG602 was inoculated in 2.5 liter transformation broth. The bioreactor was then sparged with toluene vapors to saturate the air to be utilized as biotransformation substrate. The biotransformation process was performed for 48 h at 37°C for optimum production of 3-MC. The procedures for minimization of bioreactor toxicity, extraction of the column and analysis of product were identical as 3-NC production.

## Results and Discussion

### Screening of catechol producing strains

Several strains belonging to genus *Bacillus*, *Kocuria, Ralstonia, Micrococcus *and *Pseudomonas *were obtained from the enrichment culture techniques. These isolated organisms were tested for their ability to convert 3-NP and toluene into 3-substituted catechol including wild type strain *P. putida *F1 and *E. coli *expression clone (pDTG602). Among the screened organisms only strain F1 and expression clone pDTG602 were found to be metabolizing 3-NP and toluene respectively. The organism F1 utilizing 3-NP as sole source of carbon and energy. However the expression clone (pDTG602) only transformed toluene into 3-MC in presence of growth supporting additional carbon source glucose. The selected strains were also tested for chromogenic assay and found to be strongly positive for the presence of 3-substituted catechols in the degradation pathway of 3-NP and toluene.

**Figure 2:**
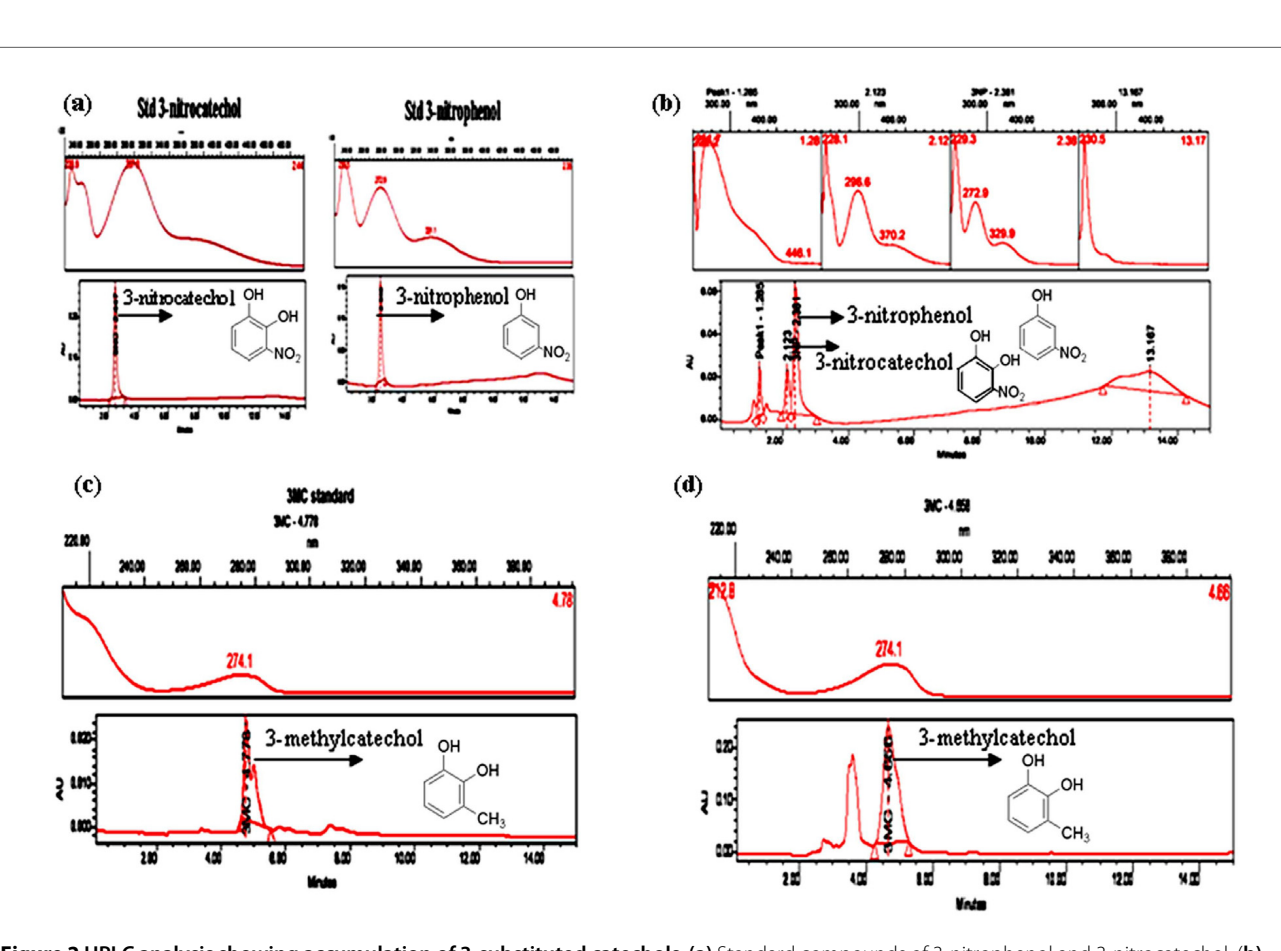
**HPLC analysis showing accumulation of 3-substituted catechols**. **(a) **Standard compounds of 3-nitrophenol and 3-nitrocatechol. (**b) **Minimal medium containing 3-nitrophenol showing accumulation of 3-nitrocatechol in the medium by strain *P. putida *F1. **(c) **Standard compound of 3-methylcatechol. **(d) **Minimal medium containing toluene showing accumulation of 3-methylcatechol in the medium by *E. coli *expression clone (pDTG602).

The organism *P. putida *F1 harbors a toluene dioxygenase (*Tod *C1C2BAD) genes, which has broad substrate activity that permits it to oxidize 3-NP and accumulate 3-NC in the growth medium (Figure 2a and 2b). On the other hand the toluene metabolizing recombinant *E. coli *expression clone (pDTG602) which harbors first two genes of toluene degradation pathway such as toluene dioxygenase and *cis*-toluene dihydrodiol dehydrogenase from native strain *P. putida *F1 cloned under regulation of a strong IPTG inducible promoter can accumulates 3-MC in the growth medium using toluene as biotransformation substrate (Figure 2c and 2d).

### Process optimization

To optimize the biotransformation processes at pilot scale certain conditions such as medium composition, amount of nutrients, pH, temperature, aeration, inoculums size, substrate concentration, culture volume, single and two step biotransformation processes were optimized at shake flask scale for optimum production of 3-substituted catechols. The optimized conditions available from the shake flask (250 ml) were tested at (2.5 liter) pilot scale in order to obtained high yields of the target compounds.

### Optimization of different conditions

**Figure 3:**
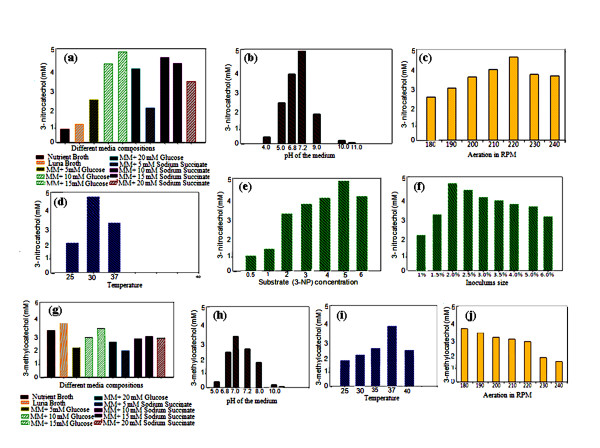
**Optimization of different conditions at laboratory scale shake flask (250 ml)**. (a) Different media compositions used for the production of 3-nitrocatechol from 3-nitrophenol by strain *P. putida *F1. (b) pH of the medium. (c) Aeration. (d) Temperature. (e) 3-nitrophenol concentration. (f) Inoculums size. (g) Different media compositions used for the production of 3-methylcatechol from toluene by *E. coli *expression clone (pDTG602). (h) pH of the medium. (i) Temperature. (j) Aeration.

Different growth media, growth supporting carbon sources at variable concentration and other important parameters were tested for maximum production of 3-NC and 3-MC. MM containing 15 mM glucose, slightly alkaline pH (7.5) and 30°C temperature was found to be optimized growth conditions for increased rate of accumulation of 3-NC in the biotransformation medium using strain *P. putida *F1. However, poor product yields were obtained with growth medium NB, LB and MM containing 5 mM or 20 mM glucose (Figure 3a,b,c,d,e and 3f). The acidic and strongly alkaline pH, lower and higher the optimum temperatures also resulted in non-optimal product recovery. Similarly, the expression clone (pDTG602) gave good yields of 3-MC in LB medium at neutral pH and 37°C incubation temperature. However, slow growth and low rate of product accumulation were observed with growth medium NB and MM containing 10 mM of glucose. Other important culture parameters e.g. effect of aeration, substrate concentration and inoculum size were also analyzed for their effect on transformation efficiency. Aeration at 220 rpm, 2% v/v inoculum size and 5 mM concentration of 3-NP were found to be optimum for 3-NC production by *P. putida *F1. On the other hand an aeration at 180 rpm and 2% v/v inoculum size was found to be optimum for 3-MC production from toluene by recombinant *E. coli *expression clone (pDTG602) (Figure 3g,h,i and 3j). Concentrations higher than 5 mM were found to be inhibitory for optimal transformation and inoculum size greater than 2% v/v adversely effected the production of the desired substituted catechol. The inhibition of transformation with higher substrate concentrations could be possibly explained on the basis of toxic effects, inhibition of the growth and metabolic activity by increased concentration of the transformation substrates [24]. Similarly, higher inoculums densities might be reduced transformation efficiency because of inefficient enzyme production due to the nutrient limitations [25].

### Effect of induction on transformation ability

**Figure 4:**
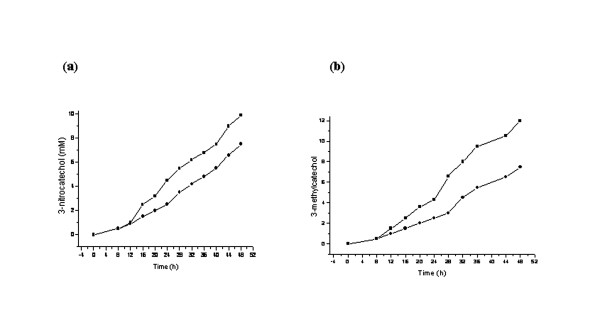
**Induced and un-induced conditions for production of 3-substituted catechols**. (a) Production of 3-nitrocatechol from 3-nitrophenol by strain *P. putida *F1 under induced (--■--) and un-induced (--●--) conditions. (b) Production of 3-methylcatechol from toluene by *E. coli *expression clone (pDTG602) under induced (--■--) and un-induced (--●--) conditions.

In order to obtain the maximum yields of the 3- substituted catechols the induction studies on bio-catalytic transformation were performed. The parameters available from preliminary process optimization experiments at laboratory scale were implemented for designing bioreactor level (2.5 liter) experiment. Interestingly, the rate of conversion of the substrates significantly increased from ~ 75% to ~95% and high yields of of 3-NC (10 mM) and 3-MC (12 mM) were obtained with pre- induced cells as compared to the un-induced cells (Figure 4a and 4b). This may be due to the fact that various physiological adaptations might be associated upon initial exposure to substrate and once adapted cells can transform the substrate rapidly at optimal level production of target compounds [24,26].

### Downstream extraction

Downstream extraction process was developed to enhance the recovery as well as purity of 3-NC and 3-MC from the biotransformation medium. Various solvents such as ethyl acetate, diethyl ether and octanol were tested for recovery of the final transformed products. The solvent octanol was found to be the suitable for maximum % recovery of the substituted catechols extraction. The results indicated that ~90% recovery of 3-NC and up to ~95% of 3-MC were obtained using octanol solvent during the process. However, the poor recovery of the products were obtained in ethyl acetate 60% of 3-NC; 70% of 3-MC. Similarly 40% recovery of 3-NC and 35% of 3-MC were obtained using solvent diethyl ether. The mass - fragmentation analysis of transformed product revealed that molecular ions peak at m/z 155* (100) (m^+1^) 125 (31), 109 (25), 81 (51), 55(61) and 53 (87) and m/z 124* (100), 123 (30), 106(9), 78 (40), 77(10), 51(9) and 39(11), which corresponds to 99% pure authentic standard of 3-NC and 3-MC respectively. The transformed products dissolved better in octanol than the other solvents and therefore, it was used for subsequent downstream extraction experiments. The optimized conditions/parameters available from the laboratory scale indicated the accuracy and applicability of 3-substituted catechol at pilot scale. Therefore, the down stream process developed during the present study is a cost effective method and has potential to produce maximum yields of the substituted catechols at pilot level.

### Qualitative and quantitative analysis

In order to check the quality and quantity of the transformed products 3-NC and 3-MC TLC study was initially performed using authentic standards of 3-substituted catechols. The Rf values of the samples drawn at different time intervals were exactly matching with standards of 3-NC and 3-MC. TLC plate was also sprayed with Folin Ciocalteu reagent an immediate development of blue coloration was apparent which indicated presence of diphenolic compounds [27]. Analysis with GC did not work well (data not shown). The possible explanation for this could be that catechols are thermally unstable and they are destroyed when subjected to high temperatures applicable in GC analysis. Amongst the three chromatographic methods HPLC performed the best for analysis the transformed products. Therefore, the formation of 3-NC and 3-MC were further confirmed by HPLC analysis. Based upon the HPLC retention time and spectral analysis the production of substituted catechols were identified at 2.123 min, 296 nm for 3-NC and 4.656 min, 274 nm for 3-MC in the spent medium during the biotransformation process.

### Pilot scale biotransformation

**Figure 5:**
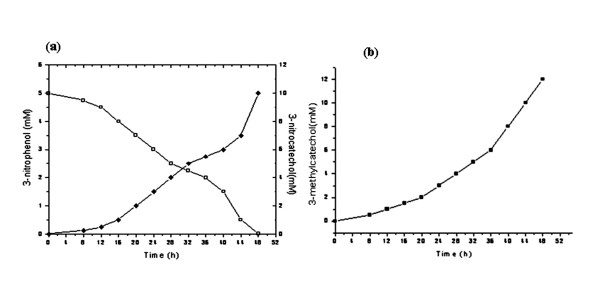
**3-substituted catechols at pilot scale**. (a) 3-nitrocatechol production (◆) with concomitant depletion of 3-nitrophenol (□) by strain *P. putida *F1. **(b) **3-methylcatechol production (■) from toluene by *E. coli *expression clone (pDTG602).

The optimized parameters were used for the pilot scale study. The samples were collected at different time intervals and analyzed in order to determine the uniformity of the process as well as to establish its applicability at pilot scale. The quantitation of all the analyzed samples of 3-NC production were clearly indicated consistent depletion of the substrate and recovery of the transformed product by applied extraction method. However, unlike the 3-NP to 3-NC accumulation, quantitative measurement of toluene depletion from HPLC was unreliable because of the high volatility of toluene. The analysis for 3-NC and 3-MC production showed the peaks of 3-NC and 3-MC after 4 h and 8 h respectively in the spent medium. However, the biotransformation process after 48 h gets plateaued and no further accumulation was observed. It is reported that high concentrations of 3-substituted catechols toxic and may cause uncoupling of NADH conversion leading to the formation of hydrogen peroxide [7,28]. Toxic effect of 3-substituted catechols on the growth of biocatalytic strains was measured at different time intervals. The results were indicated that specific growth rate sharply decreased by increasing the concentrations of 4.8 mM of 3-NC and 3.5 mM of 3-MC in transformation broth and no growth was observed after 48 h. Therefore, toxicity of 3-substituted catechols accumulation at bioreactor scale was decreased by feeding the substrate at the rate constant rate of (0.12 mole/l^-1^/h^-1^) and continuously removal of the transformed products by using external adsorbent column containing resin Amberlite ™ XAD-4 attached to the bioreactors. As a result, double the yields of 10 mM of 3-NC and 12 mM of 3-MC were obtained (Figure 5a and 5b). A similar observation of production of 3-MC up to (14 mM) at laboratory scale was reported by Husken et al. [22]. However, the present study provides significantly high yield of 3-substituted catechols accumulation at pilot scale.

## Conclusion

Biological production of substituted catechols depends on the certain conditions such as substrate, bioreactor, biomass, toxicity of substrate and product, downstream processing costs etc. The effective combination of microbial strain used and appropriate process-optimized conditions offer high productivities of the substituted catechols. The process optimization for microbial production of 3-substituted catechols in this study can serve wide industrial applications.

## Competing interests

The authors declare that they have no competing interests.

## Authors' contributions

DP and JP designed the study, carried out the experiments, analyzed the experimental data and drafted the manuscript. BNT and RKJ conceived the project, coordinated it and refined the manuscript. All authors have read and approved the final manuscript.
